# Optimization and Validation of Rotating Current Excitation with GMR Array Sensors for Riveted Structures Inspection

**DOI:** 10.3390/s16091512

**Published:** 2016-09-16

**Authors:** Chaofeng Ye, Lalita Udpa, Satish Udpa

**Affiliations:** Department of Electrical and Computer Engineering, Michigan State University, East Lansing, MI 48824, USA; udpal@egr.msu.edu (L.U.); udpa@adminsv.msu.edu (S.U.)

**Keywords:** eddy current, non-destructive testing, giant magnetoresistive sensor, optimization

## Abstract

In eddy current non-destructive testing of a multi-layered riveted structure, rotating current excitation, generated by orthogonal coils, is advantageous in providing sensitivity to defects of all orientations. However, when used with linear array sensors, the exciting magnetic flux density ($B_{x}$) of the orthogonal coils is not uniform over the sensor region, resulting in an output signal magnitude that depends on the relative location of the defect to the sensor array. In this paper, the rotating excitation coil is optimized to achieve a uniform $B_{x}$ field in the sensor array area and minimize the probe size. The current density distribution of the coil is optimized using the polynomial approximation method. A non-uniform coil design is derived from the optimized current density distribution. Simulation results, using both an optimized coil and a conventional coil, are generated using the finite element method (FEM) model. The signal magnitude for an optimized coil is seen to be more robust with respect to offset of defects from the coil center. A novel multilayer coil structure, fabricated on a multi-layer printed circuit board, is used to build the optimized coil. A prototype probe with the optimized coil and 32 giant magnetoresistive (GMR) sensors is built and tested on a two-layer riveted aluminum sample. Experimental results show that the optimized probe has better defect detection capability compared with a conventional non-optimized coil.

## 1. Introduction

The detection of deep, embedded cracks in multilayer riveted structures [1,2,3,4] is a major challenge in eddy-current (EC) non-destructive testing (NDT). Ultra-low frequency excitation along with giant magnetoresistive (GMR) sensor [5] to measure the induced magnetic field directly (EC-GMR) have been used to increase penetration depth as well as guarantee a good signal to noise ratio [6,7,8].

**Figure 1:**
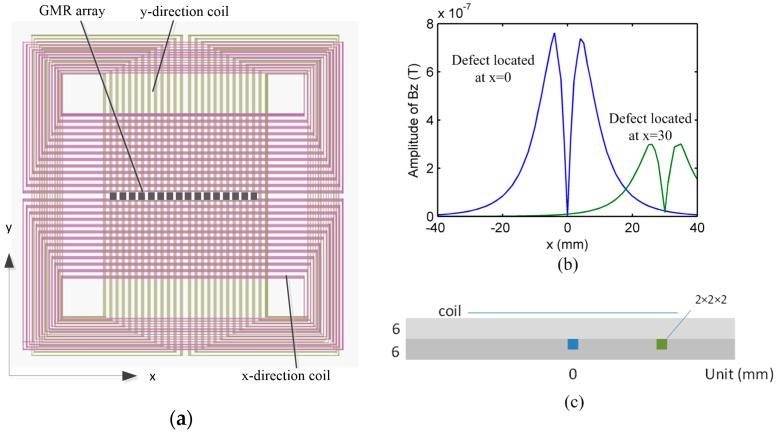
(**a**) Top view of orthogonal coils and linear array sensors; (**b**) Simulation result of response signals due to defect located at *x* = 0 mm and *x* = 30 mm; (**c**) Lateral view of the model.

The EC-GMR technique, using a single linear excitation coil, has been presented to detect fatigue cracks around fasteners in multilayer structures [9,10,11], where the induced currents are primarily generated along one direction. A linear array of GMR sensors located on the symmetry plane of the coil picks up the normal component ($B_{z}$) of a magnetic field associated with eddy currents. This design has high sensitivity when the crack is perpendicular to the current direction. However, when a crack is parallel to the EC flow, the small disturbance to eddy current flow reduces the detection capability. In order to address this problem, an excitation method that generates a rotating eddy current was presented by Yang et al. [12]. The rotating current excitation is produced by two orthogonal linear coils oriented in *x* and *y*-direction, as shown in Figure 1a. Conventionally, the wires of the coils are uniformly distributed, which is referred to as a conventional coil in the following text. The exciting current in the *x*-direction is expressed as $I_{x}=I_{0}\cos\left(\omega t\right),$ while the current in *y*-direction is $I_{y}=I_{0}\cos\left(\omega t+90{}^{\circ}\right)$, where $I_{0}$ is the current amplitude, ω angular frequency. The induced eddy current in the sample generated by the orthogonal coils is rotating in space and hence sensitive to defects in any orientation. A probe configuration with rotating current excitation has similar sensitivity to cracks emanating around fastener sites in all orientations. However, with two orthogonal coils, there is only one point of symmetry at the center of the coils. The normal component of induced flux density is zero at the symmetry point in the absence of a defect.

When using a probe with a single sensor located at the symmetry point, a 2D raster scan is needed that makes an inspection of a sample surface slow. However, if an array of sensors is used to increase inspection speed, the sensor array will be located on the symmetry plane of one coil but perpendicular to the symmetry plane of the other coil. It leads to two major issues, which give rise to some challenges in experimental implementation, namely, (1) a strong background field at sensors located off the center point and (2) non-uniform induced currents in the test sample below the sensor array location. Even theoretically, these issues can be addressed by using an extremely large size exciting coil; the total size of the probe is preferred to be small in field application. Consequently, it is necessary to make the rotating current EC-GMR probe have small size, as well as robust performance.

The differential measurement method was proposed to address the first issue of eliminating the strong background field during the scan in [13]. In this paper, the excitation coil design is optimized to generate a uniform excitation field so as to address the second issue. The performance of the optimized probe design is analyzed and compared with a conventional probe.

## 2. Conventional Uniform Exciting Coil

As shown in Figure 1a, in the rotating current configuration using orthogonal coils, the linear sensor array is located on the symmetry plane of the *x*-direction coil, and is perpendicular to the symmetry plane of the *y*-direction coil. The magnetic flux density components, $B_{x}$, generated by the *y-*direction coil, is not uniform over the sensor region, which implies that the eddy current generated by the excitation coil in the test sample is not uniform over the sensor region. 

The eddy current density at the center of the sensor array is higher in magnitude compared to the eddy current density at the edge of the sensor array at the same depth. This implies that a defect located under the sensor at the center is detected with a stronger response compared to the signal produced at the sensor when the defect is located near the coil edge at same depth. The 1D scan signals for the two cases are shown in Figure 1b, where the geometry dimensions of the model are as shown in Figure 1c. When the probe is used to inspect rows of rivets, the rivet image will be distorted due to this inconsistency. 

## 3. Exciting Coil Optimization

**Figure 2:**
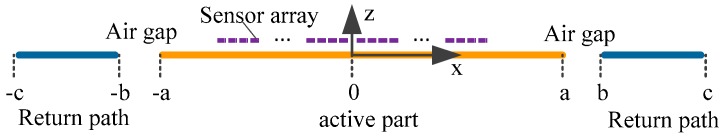
Lateral view of the *y*-direction exciting coil and sensor array.

In order to address the problems described above and improve the performance of the probe, the excitation coil design is optimized to make the excitation field uniform across the sensor array. The lateral view of the *y*-direction excitation coil and the sensor array is shown in Figure 2. The coil consists of the active region with excitation current and return paths. The active part is defined by x∈(−a,a) while the return paths lie in the interval $x\in \left(b,c\right)\cup^{\text{​}}\left(-c,-b\right)$, here 0 < *a* < *b* < *c*. 

Let $J_{\left(x\right)}$ (−*c* ≤ *x* ≤ *c*) be the current density distribution of the coil:

(1) $$J_{\left(x\right)}=J_{\left(-x\right)}$$

(2) $$\int_{0}^{c}J\left(x\right)dx=0$$

Here Equation (1) is due to the symmetric structure of the coil. The current density distribution is an even function of *x* and hence it is necessary to optimize only $J_{\left(x\right)}\;\mathrm{for}\;\left(x\geq 0\right)$. Equation (2) indicates that the total return current is equal to the total excitation current for each half of the coil to satisfy the current continuity theorem.

Since the return part is relatively far away from the sensor array and the width of the return part is kept as small as possible to make the coil size small, it is reasonable to assume that the current distribution in the return path is constant ($J_{r}$). The current density distribution in the active region is a function of *x* and needs to be optimized. Let *J*(*x*) be written as *n*^th^ order polynomial in the region (x∈[0,a]):

(3) $$J\left(x\right)=\{\begin{array}{cc} \overset{n}{\underset{k=0}{\sum }}p_{k}\;x^{k} & \;0\leq x\leq a\\ 0 & a<x<b\;\\ J_{r} & b\leq x\leq c\; \end{array}$$

Here $p_{k}\;\left(k=0,1,2,\cdots ,n\right)$ is the factor of the power series. Substituting Equation (3) into Equation (2):

(4) $$J_{r}=\frac{1}{b-c}\overset{n}{\underset{k=0}{\sum }}p_{k}\frac{a^{k+1}}{k+1}$$

Since, for riveted structure inspection, the exciting frequency is ultra-low, e.g., 100 Hz, $B_{x}$ at any observation point (*x*’, 0, *z*’) in air is written as:

(5) $$B_{x}\left(x^{\prime },z^{\prime }\right)=\int_{-c}^{c}G\left(x^{\prime }-x,z^{\prime }\right)J\left(x\right)dx$$

where G(x,z) is Green’s function expressed as [14,15]:

(6) $$G\left(x,z\right)=\frac{\mu_{0}z}{4\pi \sqrt{x^{2}+z^{2}}}\overset{\frac{l}{2}}{\underset{-\frac{l}{2}}{\int }}\frac{\mathrm{dy}}{x^{2}+z^{2}+y^{2}}$$

where l is the length of the current in *y*-direction. As the orthogonal coil is square, l=2c. Invoking Equations (1)–(5):

(7) $$B_{x}\left(x^{\prime },z^{\prime }\right)=\int_{0}^{c}[G\left(x^{\prime }-x,z^{\prime }\right)+G\left(x^{\prime }+x,z^{\prime }\right)]J\left(x\right)dx$$

Substituting from Equations (3) and (4) in Equation (7) and interchanging summation and interpolation we have:

(8) $$B_{x}\left(x^{\prime },z^{\prime }\right)=\overset{n}{\underset{k=0}{\sum }}p_{k}\left\{\int_{0}^{a}[G\left(x^{\prime }-x,z^{\prime }\right)+G\left(x^{\prime }+x,z^{\prime }\right)]x^{k}dx+\int_{b}^{c}[G\left(x^{\prime }-x,z^{\prime }\right)+G\left(x^{\prime }+x,z^{\prime }\right)]\frac{1}{b-c}\frac{a^{k+1}}{k+1}dx\right\}$$

We define:

(9) $$\gamma_{k}\left(x^{\prime },z^{\prime }\right)=\int_{0}^{a}[G\left(x^{\prime }-x,z^{\prime }\right)+G\left(x^{\prime }+x,z^{\prime }\right)]x^{k}dx+\int_{b}^{c}[G\left(x^{\prime }-x,z^{\prime }\right)+G\left(x^{\prime }+x,z^{\prime }\right)]\frac{1}{b-c}\frac{a^{k+1}}{k+1}$$

such that:

(10) $$B_{x}\left(x^{\prime },z^{\prime }\right)=\overset{n}{\underset{k=0}{\sum }}\gamma_{k}p_{k}$$

Suppose there are *m* observation points (${x^{\prime }}_{i},0,{z^{\prime }}_{i}$), *i* = 1, 2, ···, *m*. Define vector:

(11) $$B_{x}={\left[B_{x_{1}},B_{x_{2}},\ldots .B_{x_{m}}\right]}^{T}$$

Then Equation (10) can be written in compact form as:

(12) $$B_{x}=\gamma^{T}P$$

Here γ is a (*n* + 1) × *m* matrix that is defined as:

(13) $$\gamma \left(i,j\right)={\gamma_{i}}_{\left({x^{\prime }}_{j},{z^{\prime }}_{j}\right)}$$

**P** is the unknown power series factors vector,

(14) $$P={\left[p_{0},p_{2}\ldots p_{n}\right]}^{T}$$

To make $B_{x}$ uniform across all the observation points:

(15) $$\gamma^{T}P=V={\left[v,v\ldots v\right]}^{T}$$

where *v* is a non-zero constant value.

### 3.1. Size of Active Region “a”

Equation (15) is consistent if, and only if, the coefficient matrix γ and the augmented matrix have the same rank. The solvability of Equation (15) depends on a number of design parameters. One of the most important parameters is the size of the active region. Consider a sensor array with 32 GMR sensors located at ${x^{\prime }}_{i}=\pm \left(2i-1\right)\;\mathrm{mm}\;\left(1\leq i\leq 16\right)$. Since the defects are typically assumed to be in the second layer at a depth of 6 mm in the multiple-layer structure; it is required to have a uniform eddy current at this depth to avoid the distortion due to non-uniform excitation fields. The observation points where the excitation field is measured are selected as:

(16) $${x^{\prime }}_{i}=\{\begin{array}{cc} 0 & i=1\\ 2i-1 & 1\leq i\leq 16 \end{array}\;\mathrm{mm}$$

(17) $${z^{\prime }}_{i}=-6\;1\leq i\leq 17\;\mathrm{mm}$$

As there are 17 observation points (*m* = 17), *n* is set to be 16 to make γ a square matrix.

The width of the return path should be one quarter of the active region to guarantee that there is enough space for return wires when the coil is fabricated on a printed circuit board (PCB).

(18) $$c-b=\frac{a}{4}$$

Assume the air gap width between the active part and the return path ($w_{air}$) is 5 mm. Then

(19) b−a=5 mm

**Figure 3:**
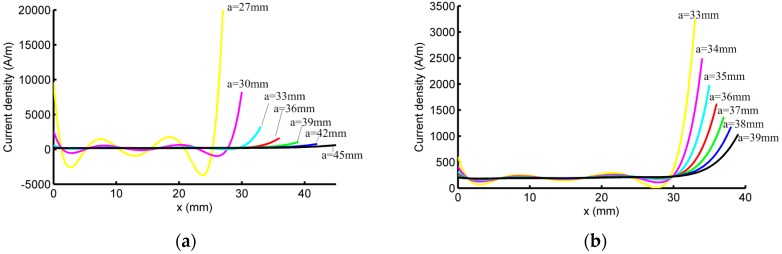
Optimization result: current density distribution vs. *x* for different values of parameter “*a*”. (**a**) 27≤ *a* ≤ 45; (**b**) 33≤ *a* ≤ 39, which is the possible range of “*a*” for optimized probe.

The effect of “*a*” is studied numerically by sweeping parameter “*a*” from 25 mm to 50 mm at increments of 1 mm. For each value of “*a*”, “*b*” and “*c*” are calculated according to Equations (18) and (19), γ is calculated from Equations (9) and (15) is analyzed. It is found that Equation (15) is not consistent when *a* ≤ 26 mm. When *a* ≥ 27 mm, a non-zero solution of **P** is derived from Equation (15) from which J(x) is calculated using Equation (3). Some of these plots of J(x) are presented in Figure 3a. It is seen that the plot of J(x) oscillates between positive and negative values when *a* ≤ 32 mm. So “*a*” should be no less than 33 mm, which is reasonable considering that the sensor array is located at x∈(−31, 31). Figure 3b presents the optimized current density distribution when 33 ≤ *a* ≤ 39, in which case J(x) is always positive.

Smaller values of “*a*” are preferred, since the overall probe size is preferred to be small. However, it should be noticed that when “*a*” is small, the variation of J(x) is large, e.g., when *a* = 33 mm, the maximum current density is 3248 A/m, while the minimum current density is 3.78 A/m. Such a distribution of J(x) cannot be realized on a PCB. So a constraint is introduced as follows:

(20) $$\eta =\frac{\max\left|J\left(x\right)\right|}{\min\left|J\left(x\right)\right|}$$

**Figure 4:**
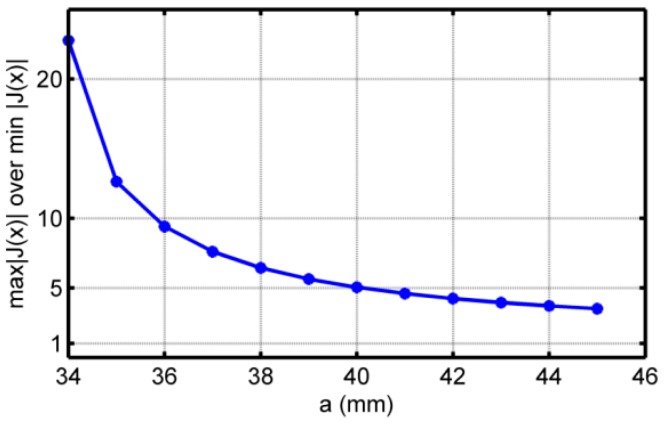
Calculation results: η vs. parameter “*a*” for fixed air gap.

Keeping fixed $w_{air}$, the variation of η vs. “*a*” is plotted in Figure 4. For η to be less than 10, we need a≥36 mm.

### 3.2. Effect of Air Gap Width “$w_{air}$”

**Figure 5:**
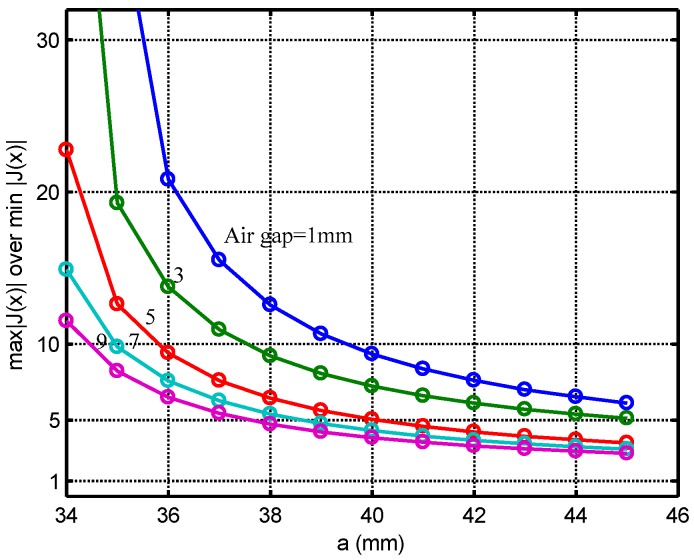
Calculation results: η vs. parameter “*a*” for different air gap width $w_{\mathrm{air}}$.

The effect of the width of the air gap between the active region and the return path ($w_{air}$) is studied in this section. $w_{air}$ is varied from 1 mm to 9 mm at increments of 1 mm. The value of η defined in Equation (20) is calculated for different values of $w_{air}$. The plots of η vs. “*a*” for different values of $w_{air}$ are presented in Figure 5. 

For each value of $w_{air}$, there is a minimum possible “*a*” (a_min_) to satisfy η<10, as listed in Table 1. The total coil size (w) can be calculated according Equation (21).

(21) $$w=2.5a+2w_{air}$$

As the air gap increases, the value of “a_min_” decreases, hence a good tradeoff is provided by allowing air gap = 5 mm, and *a* = 36 mm.

### 3.3. Coil Design

Using $w_{air}=5$ mm, *a* = 36 mm, the width of each line of the coil $d_{w}$ is derived.

(22) $$d_{w}\left(x\right)=\frac{1}{J\left(x\right)}\times \frac{\max\left|J\left(x\right)\right|}{d_{m}}$$

where $d_{m}$ is the minimum wire width, that is decided by the minimal wire clearance distance of PCB fabrication processing and the required copper wire width of carrying the exciting current. Here we use $d_{m}=0.25\;\mathrm{mm}$. The positions of each line/wire $x_{w_{i}}$ are derived according to the following equations.

(23) $$x_{w_{1}}=\frac{d_{w}\left(0\right)}{2}$$

(24) $$x_{w_{i+1}}-x_{w_{i}}=d_{w}\left(\frac{x_{w_{i+1}}+x_{w_{i}}}{2}\right),\;i>1\;\mathrm{and}\;x_{w_{i+1}}<a$$

**Figure 6:**
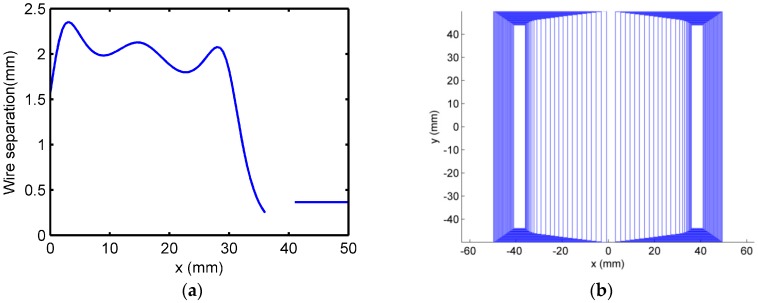
Optimized coil design 50 turns non-uniform distributed wires: (**a**) wire separation ($d_{w}$ ) vs. *x* and (**b**) top view of the optimized coil.

The final design of optimized exciting coil with 50 turns, non-uniform distributed, wires is presented in Figure 6.

## 4. Model Based Performance Analysis

A 3D finite element model (FEM) is used to analyze the performance of the optimized coil and compare the results with those obtained using conventional coil. The formulation is based on reduced magnetic vector potential formulation [16,17,18], which avoids re-meshing of coil for each position during scanning. Commercial finite elements simulation software COMSOL Multiphysics (COMSOL, Inc., Palo Alto, CA, USA) with Matlab LiveLink is used to build the model and do numerical calculation.

**Figure 7:**
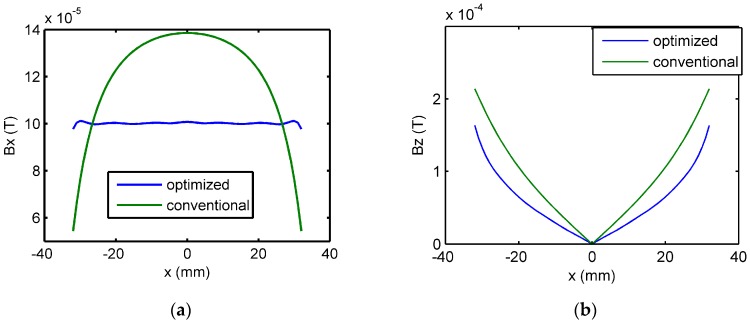
Numerical simulation results of (**a**) $B_{x}$ at the observation points and (**b**) $B_{z}$ at the sensor array for the cases of optimized coil and uniform coil.

The magnetic fields in air generated by the optimized coil and conventional coil are calculated. The two coils have identical size and number of turns. A current of frequency 100 Hz is used as excitation input. The frequency of 100 Hz is chosen to have adequate skin depth to penetrate the sample thickness. It is seen from the results presented in Figure 7a that the variation of $B_{x}$ in the sensor region is greatly reduced in the case of the optimized coil in comparison to that obtained using a conventional coil. Although the normal component magnetic field ($B_{z}$) is not an objective function during this optimization, as seen in Figure 7b, the distribution of the $B_{z}$ also becomes relatively flatter with the optimized coil, in comparison to that obtained with a uniform coil.

### 4.1. Detection of Subsurface Defects at Different Locations

**Figure 8:**
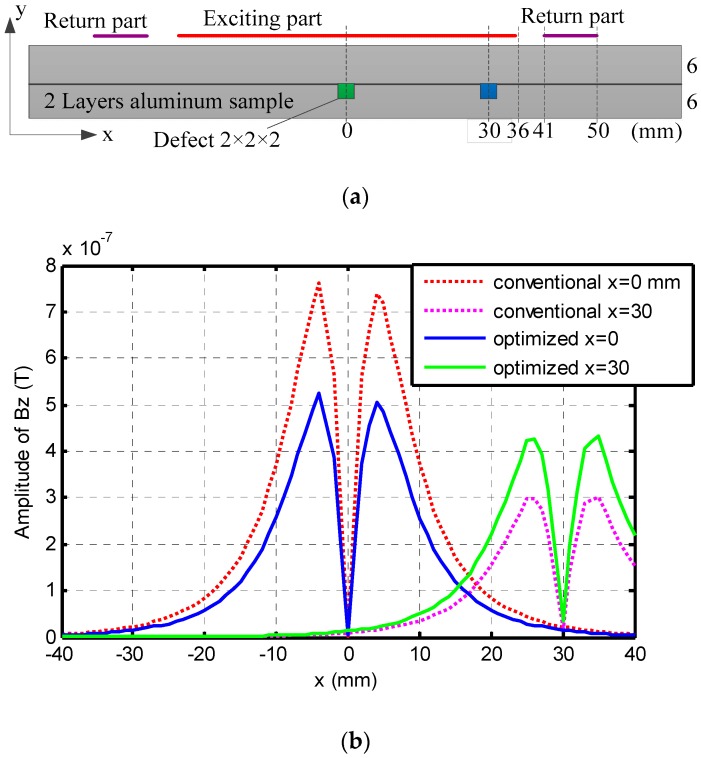
(**a**) Lateral view of tested sample and excitation coil; (**b**) Simulation results of the optimized coil and uniform distribution coil for a defect located at *x* = 0 mm and *x* = 30 mm after background subtraction.

Subsurface defects located at the center of the coil (*x* = 0 mm) and located off center (*x* = 30 mm) are simulated. The sample and defect geometry dimensions are specified in Figure 8a. Both optimized coil and conventional coil are simulated. A normal component magnetic field ($B_{z}$) 1 mm above the coil is measured. To highlight the defect signal, a background signal is obtained by placing the coil on top of a defect free aluminum sample and subtracted from the measured signals. The simulation results are presented in Figure 8b. For conventional uniform distribution coil, the peak signal is reduced to 39.23% when the defect is moved from the center of the coil to an off-center location at *x* = 30 mm demonstrating that a defect located near the coil edge is detected with a much weaker response compared to the signal produced by the same defect at the coil center. However, with the optimized coil, the peak signal only reduced to 81.25%. In the model based study, the exciting current was kept constant, in which case the peak signal of the defect located under the center of the optimized coil is smaller than that under the center of the conventional coil; but it doesn’t impact defect detectability of the sensor since the signal amplitude can be easily increased by using a larger exciting current. In practice, exciting current amplitude is tuned to make the strongest signal almost equal to the measurement range to obtain best sensitivity. In summary, it is seen from the simulation result that the optimized coil has better performance for detecting a defect located under the edge of the sensor array.

### 4.2. Detection of Defect under Fastener Head

**Figure 9:**
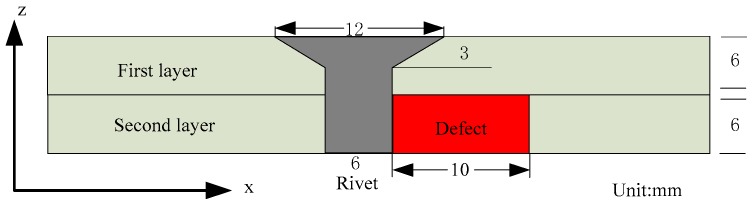
Geometry of the test sample used in the finite element model (FEM) model.

Next, defect and fastener geometry is modeled using the finite element model. The test specimen is a two-layered aluminum structure as shown in Figure 9. A radial notch spans the thickness of the second layer. Rotating current excitation with two identical orthogonal coils is used in these simulations. The excitation frequency is 100 Hz and current amplitude is 1 A. The orthogonal coils are located at a 1 mm liftoff from the top surface of the test sample. A C-scan image is produced by calculating the normal component of the magnetic field ($B_{z}$) values at a 1 mm liftoff distance from the coils at each scan point. To highlight the rivet and defect signal, the background signal obtained by placing the coils on top of defect free aluminum sample is subtracted from the measured signal.

**Figure 10:**
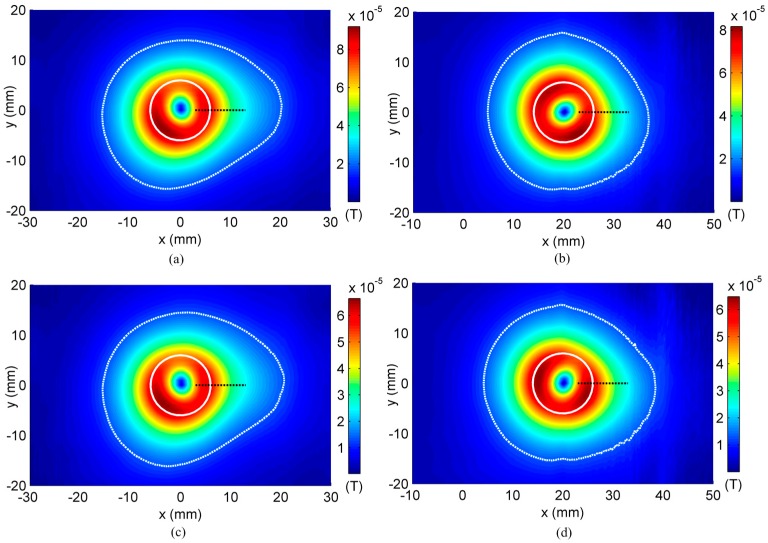
Simulation results for cases: (**a**) conventional coil, rivet center located at *x* = 0 mm; (**b**) conventional coil, rivet center located at *x* = 20 mm; (**c**) optimized coil, rivet center located at *x* = 0 mm; (**d**) optimized coil, rivet center located at *x* = 20 mm.

Two different cases are simulated: (1) the rivet center is aligned with the orthogonal coils’ center (*x* = 0); (2) the rivet center is shifted by 20 mm along *x* axis (*x* = 20). The simulation results showing magnitude of the normal component magnetic field ($B_{z}$) is calculated and plotted in Figure 10. The improved performance of the optimized coil is seen visually in the C-scan image. The improvement in performance can be quantitatively estimated as follows.

**Figure 11:**
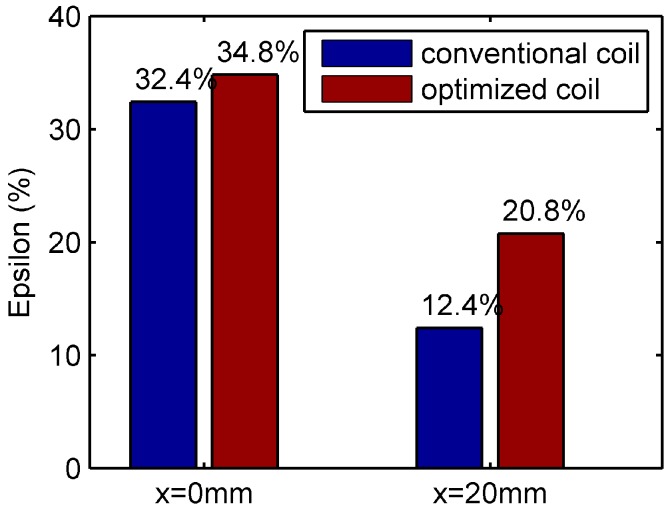
Simulation Results—Comparison of reduction in ϵ of the conventional coil and optimized coil for centered and off-center rivets.

To quantify the distortion introduced by the presence of a defect, all contiguous pixels above a preselected threshold value v are identified and a contour plot enclosing the region containing pixels is drawn. The contour plot obtained by setting v equal to 30% of the peak value is plotted in Figure 10. Defining the deformation coefficient ϵ as [13]:

(25) $$\epsilon =\left|\frac{r_{max}-r_{0}}{r_{0}}\right|\times 100\%$$

where $r_{0}$ is the radius of the contour plot of the image obtained with a defect free rivet. $r_{\max}$ is the maximum distance from the rivet center to the contour plot. The deformation coefficient is a simple straightforward parameter for evaluation of the distortion of the signal in a rivet area. Some other more complex algorithms may be applied. In fact, our lab colleagues tried different methods. For example, J. Kim et al. used principal components analysis to classify the data [19], a multi-frequency mixing algorithm was utilized for the suppression of the rivet signal [20]. As this paper is mainly about sensor optimization, the deformation coefficient is used to quantize the signal. The deformation coefficients of the simulation results are compared in Figure 11. 

It is found that when the rivet center is shifted by 20 mm from the coil center along *x* axis, the distortion coefficient for uniform distributed coil drops from 32.42% to 12.41%. However, ϵ for the optimized coil only reduces from 34.8% to 20.8%. The optimized coil has better defect indication when the rivet and defect are misaligned with the coil center.

## 5. Experimental Validation

### 5.1. Multi-Layer Coil Design

**Figure 12:**
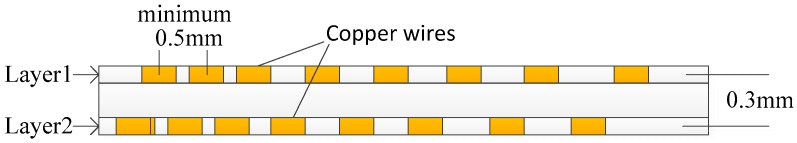
Schematic of the lateral view of the multi-layer coil design.

A prototype probe with the optimized coil is fabricated and instrumented to test the performance of the proposed design. The multi-layer coil is fabricated using a multi-layers print circuit board (PCB). A novel coil design, named multi-layer coils (MLC), is proposed to fabricate the optimized coil. Each coil consists of at least two layers. Coppers from different layers stagger to cover the air gaps, as shown in Figure 12, so that the current distribution is more continuous in comparison with that of a single layer coil. 

### 5.2. Experiment Setup

**Figure 13:**
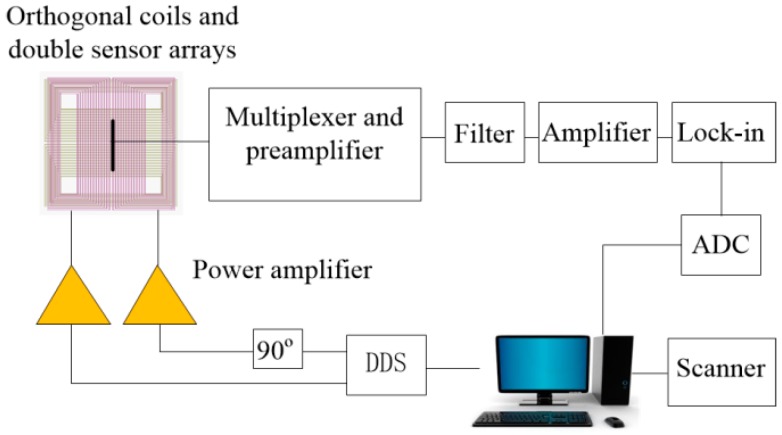
Block diagram of experiment system.

The experimental system is shown schematically in Figure 13a. GMR Sensor Model GF708 from Sensitec (Lahnau-Waldgirmes, Germany) is used to build the prototype. A linear sensor array with 32 sensors is used. The distance between two sensors is 1.6 mm. The normal component of the magnetic flux density $B_{z}$ is measured by the array sensors. The sensor array is located on the symmetry plane of the *x*-direction coil, 1 mm above the coil.

A sinusoidal current of amplitude 150 mA and frequency 100 Hz is applied to the two orthogonal coils with a 90° phase difference. A lock-in amplifier model RF840 from Stanford Research System is used to obtain the baseband signal with the best possible signal to noise ratio (SNR). The output of the lock-in amplifier is sampled and stored. The experimental measurements are obtained using a high resolution scanning system.

### 5.3. Experimental Results and Discussion

**Figure 14:**
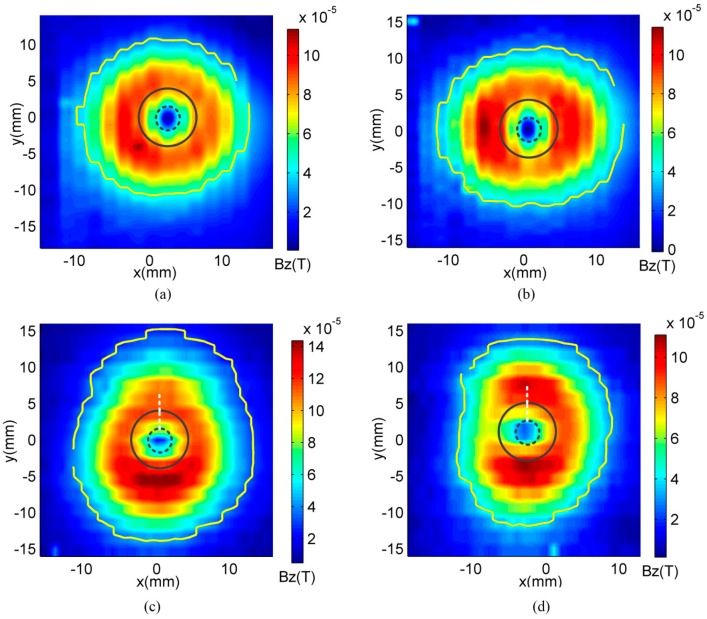
Experiment results of (**a**) defect free rivet generated using optimized coil; (**b**) defect free rivet generated using conventional coil; (**c**) rivet with a 6 mm length 2nd layer defect generated using optimized coil; (**d**) rivet with a 6 mm length defect generated using conventional coil.

The performance of the optimized probe is compared with that of a conventional coil for the cases of the rivet located at *x* = 20 mm off the coils’ center. A defect free aluminum fastener and a fastener with a 6 mm length defect in the second layer are scanned. The experimental results are presented in Figure 14, in which the contour selected at 30% of the maximum value in the image is plotted. Theoretically, the image of the defect free rivet should be circular [12], as seen in Figure 14a. However, it is seen from Figure 14b that the image of the defect free rivet generated using the conventional coil is distorted due to the non-uniform distribution of the excitation field and eddy currents. 

**Figure 15:**
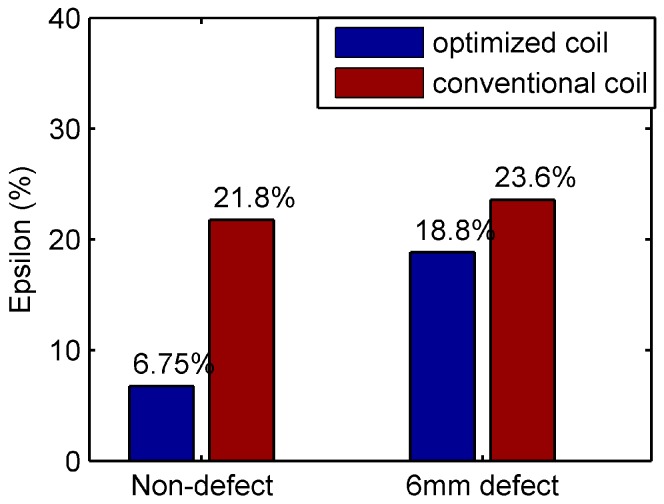
Experimental Results—Comparison of reduction in ϵ of the conventional coil and optimized coil for centered and off-center rivets.

The deformation coefficient ϵ defined in Equation (25) is calculated to quantify the performance, as shown in Figure 15. For optimized coil, ϵ is 6.75% for the defect free rivet and increases to 18.8% for the rivet with a 6 mm length defect. However, for the conventional coil, ϵ changes from 21.8% to 23.6% for the defect free and defective rivet. The optimized coil has better defect detection capability compared with the conventional coil.

## 6. Conclusions

In this paper, the current distribution of rotating current excitation is optimized with respect to a linear sensor array. A non-uniform coil design was derived to generate the optimized current distribution. The main desirable feature of the optimized coil design is that the excitation field is more uniform in the sensor array region than that of the conventional coil.

The performance of the optimized design was studied numerically based on FEM models and validated experimentally using a prototype probe to test a riveted multi-layer aluminum sample. Simulation results show that for the conventional coil, a subsurface defect located near the coil edge is detected with a much weaker response compared to the signal produced when the defect is located at the coil center. However, the reduction in signal magnitude is much less in the case of an optimized coil. For the inspection of a defect located under the rivet head, the distortion coefficient of the optimized coil is greater than that of the uniform coil when the fastener and defect are not aligned with the coils’ center. 

A new multi-layer coil structure was proposed to fabricate the optimized coil of the prototype probe. Experiment results demonstrate that the optimized probe has a higher detection capability of a subsurface defect in a multi-layer structure compared to a conventional coil.

## Figures and Tables

**Table 1:** Minimum coil size satisfying η<10 for different air gap width.

Air Gap (mm)	1	2	3	4	5	6	7	8	9
“a_min_” (mm)	40	39	38	37	36	36	35	35	35
w (mm)	102	101.5	101	100.5	100	102	101.5	103.5	105.5
